# Optically Resonant Bulk Heterojunction PbS Quantum Dot Solar Cell

**DOI:** 10.1021/acsnano.1c11330

**Published:** 2022-08-29

**Authors:** Stefan W. Tabernig, Lin Yuan, Andrea Cordaro, Zhi Li Teh, Yijun Gao, Robert J. Patterson, Andreas Pusch, Shujuan Huang, Albert Polman

**Affiliations:** † Center for Nanophotonics, NWO-Institute AMOLF, Science Park 104, 1098 XG Amsterdam, The Netherlands; ‡ School of Photovoltaic and Renewable Energy Engineering, University of New South Wales, 229 Anzac Parade, 2052 Sydney, Australia; § Van der Waals-Zeeman Institute, Institute of Physics, University of Amsterdam, Science Park 904, 1098 XH Amsterdam, The Netherlands; ⊥ School of Engineering, Macquarie University, Sydney 2109, Australia

**Keywords:** bulk heterojunction, light trapping, quantum dot solar cells, charge-carrier extraction, nanoimprint, generation profiles, optoelectronic enhancement

## Abstract

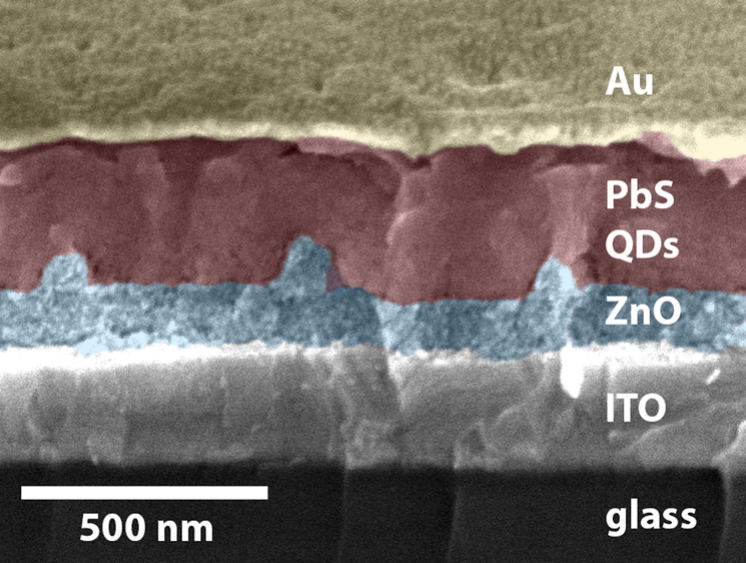

We design an optically resonant bulk heterojunction solar cell to study optoelectronic properties of nanostructured p–n junctions. The nanostructures yield strong light–matter interaction as well as distinct charge-carrier extraction behavior, which together improve the overall power conversion efficiency. We demonstrate high-resolution substrate conformal soft-imprint lithography technology in combination with state-of-the art ZnO nanoparticles to create a nanohole template in an electron transport layer. The nanoholes are infiltrated with PbS quantum dots (QDs) to form a nanopatterned depleted heterojunction. Optical simulations show that the absorption per unit volume in the cylindrical QD absorber layer is enhanced by 19.5% compared to a planar reference. This is achieved for a square array of QD nanopillars of 330 nm height and 320 nm diameter, with a pitch of 500 nm on top of a residual QD layer of 70 nm, surrounded by ZnO. Electronic simulations show that the patterning results in a current gain of 3.2 mA/cm^2^ and a slight gain in voltage, yielding an efficiency gain of 0.4%. Our simulations further show that the fill factor is highly sensitive to the patterned structure. This is explained by the electric field strength varying strongly across the patterned absorber. We outline a path toward further optimized optically resonant nanopattern geometries with enhanced carrier collection properties. We demonstrate a 0.74 mA/cm^2^ current gain for a patterned cell compared to a planar cell in experiments, owing to a much improved infrared response, as predicted by our simulations.

With the continuing rise of photovoltaics (PV) as one of the main pillars of our current and future energy supply system, more and more PV-related applications have appeared and grown into important subfields such as building-integrated PV,^1−3^ vehicle-integrated PV,^4^ and PV-integrated electronic devices.^5^ While Si solar panels represent the main markets for rooftop solar and large-scale PV plants for the near future, many important high-value markets desire PV materials with higher flexibility. Colloidal PbS quantum dot (QD)-based absorbers have emerged as interesting contenders for applications beyond the established large-scale Si PV market. The fact that their bandgap can be tuned across a wide range of the AM1.5G solar spectrum by changing their size^6^ makes them an attractive material for various applications beyond traditional PV, such as flexible PV,^7^ luminescent solar concentrators,^8,9^ multijunction PV cells,^10,11^ and other optoelectronic applications.^12^ Furthermore, PbS QD based solar cells can be fabricated by solution processing, which makes them highly relevant for solution deposition methods such as printing via slot-die coating^13^ or spray-coating,^14^ enabling high-speed low-cost manufacturing. Earth-abundance of PbS itself and the recent power conversion efficiency record of 13.8%^15^ underline the relevance and promise of PbS QD absorber-based PV.

One major obstacle that has prevented PbS QD solar cells from keeping up the pace with, for example, perovskite PV is the short carrier diffusion length, induced by the nature of PbS QD absorbers. As they are composed of a matrix of closely packed nanocrystals with ligands as spacers,^16^ charge transport involves a hopping mechanism.^16^ The resulting short diffusion length leads to an unsatisfying compromise between optical performance (high short-circuit current, *J*_*SC*_), which requires a thick absorber layer, and electronic performance (high open-circuit voltage, *V*_*OC*_; high fill factor, *FF*), which is best for thin layers. So far, approaches to increase the diffusion length have involved reduction of QD–QD separation inside the matrix using shorter ligands,^17^ utilizing stronger electric fields inside the cell via improved device architectures,^18,19^ reduction of defect densities via better surface passivation,^17^ and reduction of average carrier-transport layer distances via bulk heterojunctions.^20,21^ Furthermore, improving the absorption of the active layer with front or backside light trapping layers such as backreflectors,^22^ slightly dented electron transport layers (ETL),^23^ bulk-heterojunctions,^24,25^ patterned ITO substrates,^26^ and on a larger scale, pyramidal ETL^27^ have all been investigated.

With some exceptions,^27−29^ these optimizations were made mainly considering either charge extraction or charge generation, but not both. Here, we introduce a nanostructured heterojunction geometry that both optimizes charge collection and serves as an optically resonant light absorber at the same time. It comprises an architecture in which the heterojunction itself is shaped as a resonant optical cavity. We present this concept by shaping colloidal PbS QD layers as absorber material with local optical resonances and guided (plasmonic) modes.

In the following we will first introduce the concept of an optically resonant p–n junction and then will show how the structure’s dimensions are chosen to maximize the absorption per unit volume, using optical simulations. This is followed by an investigation of the electronic performance of the optically optimized structure, using simulations of the carrier diffusion and collection pathways in the nanopatterned cells. We derive a nanopatterned geometry that shows a strongly enhanced optical absorption spectrum with a heterojunction geometry that strongly enhances carrier collection of charges generated at the bottom of the absorber. We use direct soft imprint process to create the nanopatterned electron conducting ZnO layer. Experimentally measured external quantum efficiency spectra confirm the enhanced light absorption and carrier collection for the optically resonant heterostructure geometry.

Our results are applicable for a wide range of emergent PV materials, including organics, TiO_2_ -dye absorbers, SbSSe, and CZTS, that all suffer from limited carrier diffusion length. Our study indicates that by structuring the p–n junction in three dimensions, the constraints imposed by short diffusion lengths can be weakened, if care is taken with respect to where carriers are generated, as well as how the electric field distribution in the cell is influenced by the nanopattern’s geometry. In practice, for each material, the optimum nanopatterning process will depend on the deposition method of the absorber and the surrounding n-/p-window or extraction layers.

## Results and Discussion

### Design and Fabrication

**Figure 1:**
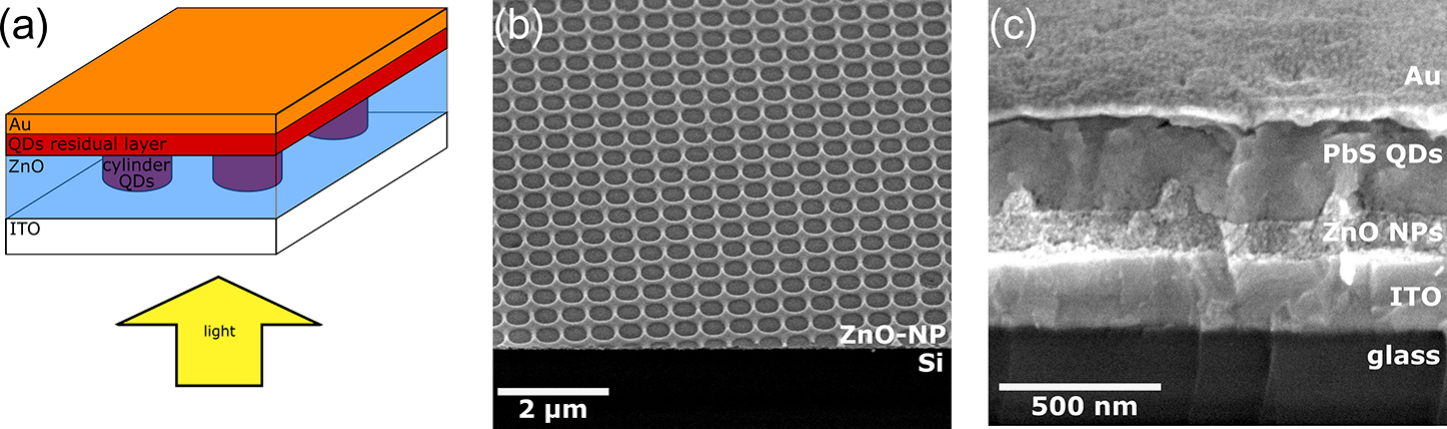
(a) Patterned p–i–n for a PbS QD solar cell heterojunction structure. Light is coming from the bottom. ITO and Au serve as front and back contacts, respectively. Bulk PbS QDs act as the p-/i-type layer, and ZnO as the n-type layer, together forming a 3D interface. (b) SEM perspective (45°) of SCIL-patterned ZnO nanoparticle layer on top of a silicon substrate. (c) SEM cross-section image of the patterned cell geometry. Light incident from the bottom. The ZnO holes have been completely infiltrated by PbS QDs and a flat Au layer caps off the geometry.

The design is based on a depleted-heterojunction PbS QD solar cell, which consists of the following layers, from top to bottom (see Figure 1a): ITO (front contact), a layer of ZnO nanoparticles (n-type window layer), bulk PbS QDs with PbI_2_ ligands (i-type absorber layer), bulk PbS QDs with EDT ligands (p-type layer; EDT: ethanedithiol), and Au (back contact). The “bulk QD layer” is a matrix of closely packed QDs. This layer is then structured into an array of nanocylinders composed of PbS QDs on a residual PbS QD layer, surrounded by ZnO, as illustrated in Figure 1a. The front and back contacts as well as the p-type layer form planar layers, meaning the nanopattern is confined to the i–n junction within the solar cell. The residual QD layer consists of a thin p-type QD layer and an i-type QD layer and functions as an electronic barrier between the n-type ZnO layer and the Au contact at the back. The dimensions of the pillars are chosen such that they show strong local optical resonances and guided (plasmonic) modes. In our design, the pillars are separated by a fixed distance and arranged in a square lattice. As we will see further on, the nanogeometry creates much shortened carrier collection paths for carriers generated at the back of the cell.

To fabricate the structure shown in Figure 1a, we first nanopattern holes into a ZnO-NP layer (light-blue) on top of ITO and second fill these up with QDs (red). For the patterning, we use substrate conformal soft-imprint lithography (SCIL^30^), which uses a PDMS stamp that contains nanostructures, that are replicated from a nanostructured Si master wafer, to imprint patterns into a liquid solgel. So far, SCIL was used to pattern optically functional silica-like layers. Here, we use high-resolution SCIL-stamps together with ZnO-NPs to fabricate a layer that is optically and electronically functional. Figure 1b shows such a patterned ZnO layer, consisting of holes of a diameter of 400 nm, arranged in a square lattice with a pitch of 513 nm. The height difference between the residual ZnO layer and the pattern’s walls is roughly 100 nm and is fixed by the feature depth in the PDMS stamp. It should be noted that this direct transfer of the nanopattern from the stamp into the ZnO-NP layer represents a straightforward approach and is much less likely to cause undesired degradation of optical or electronic properties of the ZnO layer compared to more conventional patterning schemes that involve reactive ion etching (RIE) and/or wet-etching. Additionally, the ZnO-NP solution used to fabricate the ETL layer corresponds to the state-of-the-art in the field of QD solar cells,^31^ which means that no compromise between quality of the ETL layer and compatibility of its precursor solution with SCIL had to be struck. The only parameter tuned for the ZnO-patterning was the spinning duration to make sure that the layer was still liquid enough to be patterned. High boiling point solvents such as buthanol allow for dilution of the solution and could serve as an additional fabrication parameter if thinner ZnO layers were needed, but this was not necessary in our case.

Next, the holes in the ZnO layer were filled up with PbS QD-ink. This was followed by deposition of a planar PbS QD-EDT layer of 30 and 100 nm of Au. Figure 1c shows the seamless interface between the ZnO and QD layers, highlighting the effectiveness of the hole infiltration with QD ink. It also becomes clear from the figure that the nanopattern is confined to the interface between ZnO and QDs, which means that the i-type material exhibits a planar surface morphology. Hence, conformal deposition of p-type QDs and the Au layer is possible in the same way as for a planar cell.

### Optical Optimization

Following the successful realization of QD pillars embedded in ZnO, we investigate the specific dimensions required to optimize the efficiency gain. We first study the optical properties of bulk PbS QD layers and maximize the absorption per unit volume in the patterned absorber by means of finite difference time domain (FDTD) simulations.

The optical properties of bulk PbS QD layers derive from a combination of the properties of PbS, which is a narrow bandgap semiconductor (0.41 eV^32^), the quantum confinement effect that occurs in nanocrystals with diameters smaller than the Bohr radius of electron–hole pairs,^6^ and the effective refractive index of the organic matrix that contains the individual PbS QDs and consists of the ligands that cap them. The effective medium formed by ligands and nanocrystals determines the refractive index of the bulk QD layer and is dominated by the choice of ligands.

**Figure 2:**
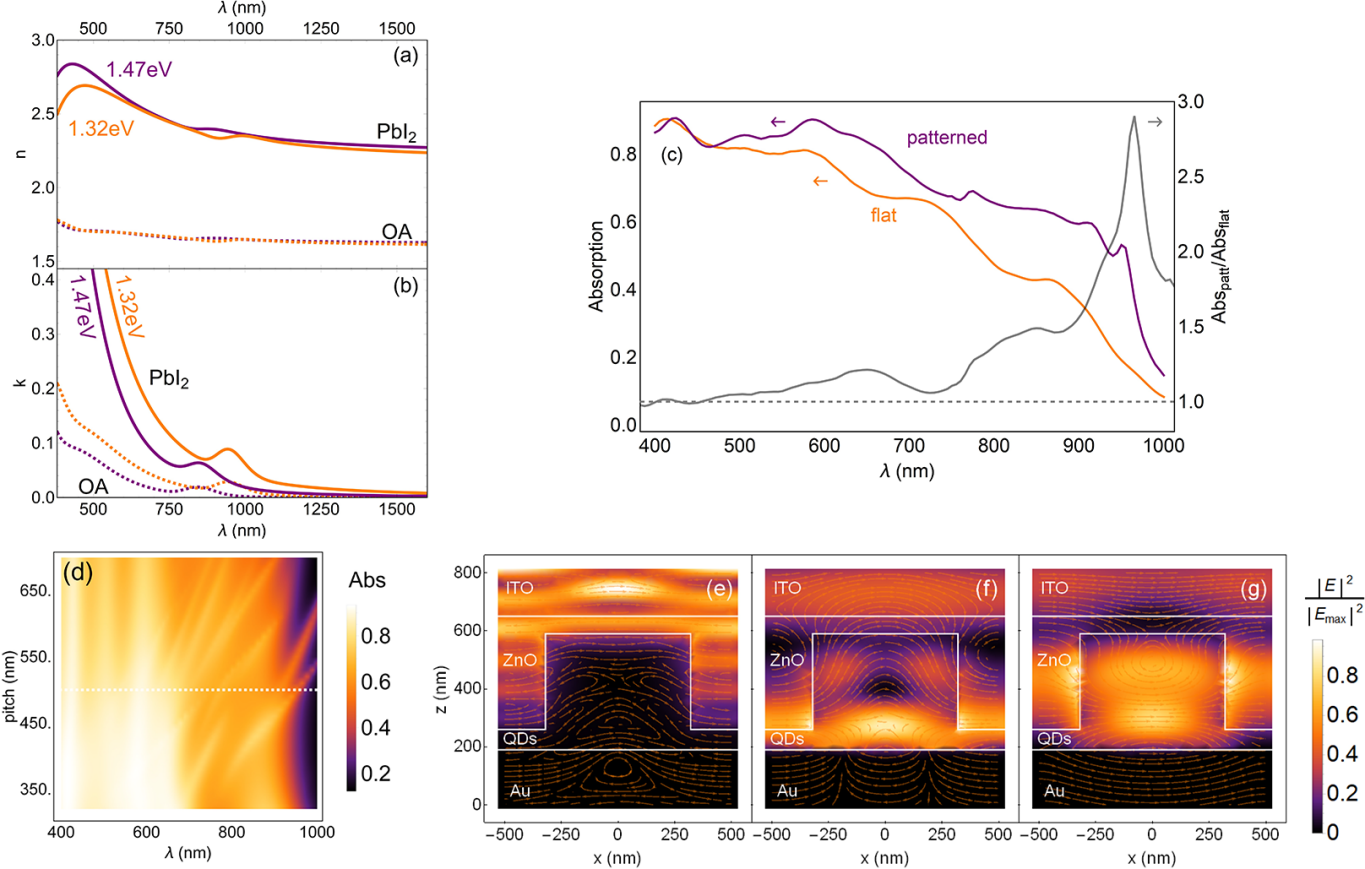
(a) Real and (b) imaginary parts of the refractive index of PbS QDs. For QDs with the first exciton peak at 1.47 eV (purple) and 1.32 eV (orange) and with either PbI_2_ (solid) or oleic acid (OA, dashed) ligands. (c) Absorption versus wavelength for a planar cell of 180 nm absorber thickness (orange) and a patterned cell that uses the same absorber volume (purple). Relative increased absorption for the patterned case is plotted on the right axis (gray line). Pillar dimensions of patterned case: 330 nm height, 320 nm diameter, 500 nm pitch. (d) Absorption versus wavelength and array pitch of the QD pillars. The color scale represents the absorption value. Data at the white dashed line correspond to the purple absorption spectrum for the patterned case in panel c. (e–g) Relative electric field intensities (color scale) across the solar cell’s cross-section through the center of a pillar for wavelengths of 425, 915, and 950 nm, respectively. White lines and labels are used to distinguish between different cell regions. Orange arrows are used to indicate the orientation of the electric field in the *x*–*z* planes.

Figure 2a and b show the real (*n*) and imaginary (*k*) parts of the refractive index of PbS QD films with different first exciton peaks and ligands, determined by spectroscopic ellipsometry (SE). We show data for first exciton peaks at 1.32 and 1.47 eV, as well as data for QDs capped with oleic acid (OA) ligands and PbI_2_. The low energy peak in *k* corresponds to the homogeneously broadened first exciton peak, which is further inhomogeneously broadened due to the size dispersion of the QDs. In general, absolute values and spectral shapes of the curves for *n* and *k* are in good agreement with literature.^33^ PbI_2_-capped QDs show a higher real part of *n* and stronger absorption compared to those with OA, which can be explained by the much denser film in the case of the short PbI_2_ ligand (3 atoms) compared to the large separations induced by the much longer OA ligand (18 C atoms^34^). The absorption for the QDs with the small bandgap (large QDs) is higher than for the large bandgap QDs. This is the case because larger QDs have a higher density of states available for absorption. The energy labels assigned to the graphs in Figure 2a represent the first exciton peak energies. The actual semiconductor bandgap of the QD layer, that is conventionally defined by a tauc-plot,^35^ is lower. In fact, the bandgap for the QDs with a first exciton peak at 1.47 eV is around 1.34 eV. This is very close to the ideal bandgap according to the detailed balance limit,^36^ and we will be using this particular set of optical constants for our optical simulations.

Using FDTD (Lumerical), we now investigate by how much the absorption per unit volume in a QD pillar can be increased by nanopatterning the layer, assuming the AM1.5G solar spectrum. We systematically vary height, diameter, and pitch of the cylinders. Furthermore, the residual QD layer thickness that separates the ZnO layer and the Au backcontact is optimized. We also considered the following boundary conditions. The residual layer is restricted to a minimum thickness of 30 nm (corresponding to 2 layers of EDT-capped QDs) and the final thickness is chosen to be 70 nm because the gain in absorption is lower for thicker layers. The pillar height was constrained to a maximum QD layer height of 400 nm, including residual layer, which corresponds to the thickness of many of the currently best performing devices.^15^ A thicker layer would suffer from large recombination losses, which makes that range less interesting for our investigation.

As figure of merit (FOM), we choose the enhancement of absorption per unit volume, defined as the ratio of the absorption within the QD volume of a patterned cell and the absorption in a planar cell with the same QD volume. Figure 2c shows the results optimized for the patterned cell and planar cell that use the same amount of QD volume. A clear enhancement in absorption is found across most of the investigated spectrum. It is largest toward the infrared range, peaking at 2.8-fold enhancement close to the bandgap and corresponds to an integrated gain of 19.5% for the spectral range considered. Overall, the spectrum shows a better IR-response due to an effectively sharper absorption onset at the bandgap energy, a mitigated dip/plateau toward the short wavelength side of the first exciton peak, as well as a more gradual increase in absorption toward the UV range, in comparison to the planar case. The difference found in the comparison of these spectra is indicative of optical enhancement beyond what one would expect from differences due to interference minima and maxima in planar solar cells.

The sharp onset of the absorption around the bandgap energy is induced by two quasi-guided modes^37−39^ in the plane of the nanopatterned layer as seen from the modal field profiles at 915 nm (f) and 950 nm (g), respectively. Toward shorter wavelengths, another sharp peak (780 nm) can be identified, also related to a quasi-guided mode. The cancellation of the dip next to the first exciton peak stems from a Fabry–Perot resonance that was placed in that wavelength range by adjusting the height of the QD pillar, effectively forming a cavity. The UV range is not significantly influenced by the pattern for two reasons. First, the dimensions obtained from the optimization are most ideal for the IR range, as there is more to gain and hence the FOM mainly is optimized within that range. Second, higher energy light is absorbed well within the first 100 nm of the QD absorber, which means that UV light does not penetrate far enough into the absorber for it to excite resonant modes in the nanostructure (Figure 2e).

To confirm the nature of the resonances, we first study the dependence of the resonant peak wavelength on the periodicity of the QD pillar lattice (Figure 2d). We find that with increasing separation of the pillars, the sharp resonances continuously shift toward longer wavelengths, which indicates that they are not a property of the QD pillar itself but a property of the pillar lattice, as determined by its periodicity (pitch). In principle, adjacent pillars can couple and exhibit collective resonances,^40^ but we rule these out as the sharp features do not vanish with increasing pillar–pillar separation, but only shift in wavelength. To investigate this further, we investigate the electric field line profiles for wavelengths of interest at the cross-section through the patterned solar cell. This analysis shows that 780 and 950 nm (Figure 2e,g) peaks correspond to quasi-waveguide modes that propagate within the complex of the structured QD–ZnO interface. Furthermore, the 915 nm peak (Figure 2f) corresponds to field line profiles that can be associated with surface plasmon polaritons that propagate at the QD–Au interface. While surface plasmon polaritons are lossy due to modal overlap with the metal, enough of the mode’s electric field distribution resides within the absorber layer to yield a net absorption gain.

### Electronic Analysis

Next, we investigate how the nanostructured geometry performs electronically by using Lumerical CHARGE, which is a drift-diffusion equation solver. We use literature data^18,25,41−45^ for energy levels, doping density, recombination, and to account for bulk QD specific properties like hopping as charge transfer mechanism and voltage loss due to the fact that an effective conduction band slightly below the conduction band is formed by defects.

In general, for planar quantum dot solar cells, the efficiency as a function of thickness peaks at an absorber thickness for which the trade-off between charge-carrier generation and extraction is at its optimum. PbS QD solar cells utilize electric field-associated drift to guide charges toward their extraction interfaces. As these electric fields only span across the depletion region within the cell, diffusion rather than drift is the dominant charge extraction mechanism for absorber layers much thicker than the depletion region. This leads to increased recombination with increasing absorber thickness.

**Figure 3:**
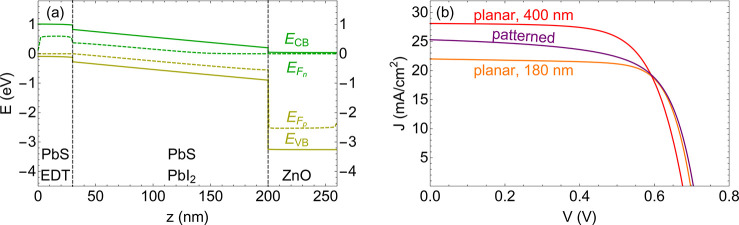
(a) Band diagram of a PbS QD solar cell (here: 180 nm absorber layer) at short-circuit current (*J*_*SC*_) condition (*V* = 0). From top to bottom: conduction band (*E*_*CB*_), electron quasi Fermi level (*E*_*Fn*_), hole quasi Fermi level (*E*_*Fp*_), valence band (*E*_*VB*_). Gray dashed lines are used to distinguish between different regions in the p–i–n junction. Light incident from the right site. (b) Simulated current density (*J*) as a function of voltage (*V*) for planar cells with absorber thicknesses of 180 nm (orange) and 400 nm (red), as well as a patterned absorber layer (purple).

An electronic band diagram of a planar cell with 180 nm absorber thickness is shown in Figure 3a (applied voltage of 0 V). The graphs can be separated into three distinct regions along the *z*-directions, corresponding to the p-, i-, and n-type layers. Band bending occurs across the full range of the i-type layer and corresponds to the presence of electric fields and is typical of depleted heterojunction solar cells.^12^ The energy offset of the p-type QD layer compared to the i-type QD layer results from the fact that dipole moments of ligands affect the energy landscape of bulk QDs. Similar conduction and valence band profiles and offsets can be found in the literature.^46^

To investigate the electronic performance of the patterned QD solar cells, we show simulated current–voltage (*I*–*V*) curves of a patterned cell and planar cells of 180 and 400 nm thickness under AM1.5G illumination in Figure 3b. The patterned cell corresponds to the set of dimensions that we obtained from the maximization of absorption per unit volume shown in Figure 2c. The thin (180 nm) planar cell uses the same amount of absorber volume as the patterned cell, and the thick (400 nm) planar cell corresponds to the thickness of QD pillar and residual layer of the absorber structure in the patterned case. Interestingly, the patterned cell shows a short-circuit current (*J*_*SC*_) that is 3.2 mA/cm^2^ larger than that of the thin planar cell, and it exhibits an open-ciruit voltage (*V*_*OC*_) that is slightly above that of the thin planar reference. The fill factor (FF) is decreased for the patterned case compared to the thin planar case. Overall, the efficiency increased by 0.4% to 11.6% in comparison to the thin reference (11.2%). Compared to the 400 nm planar reference, the patterned cell shows a slightly lower current (2.8 mA/cm^2^), an increased *V*_OC_, and a lower FF, yielding an overall lower efficiency. From these two comparisons, it becomes obvious that the main obstacle for a higher efficiency of the patterned cell appears to be the FF.

**Figure 4:**
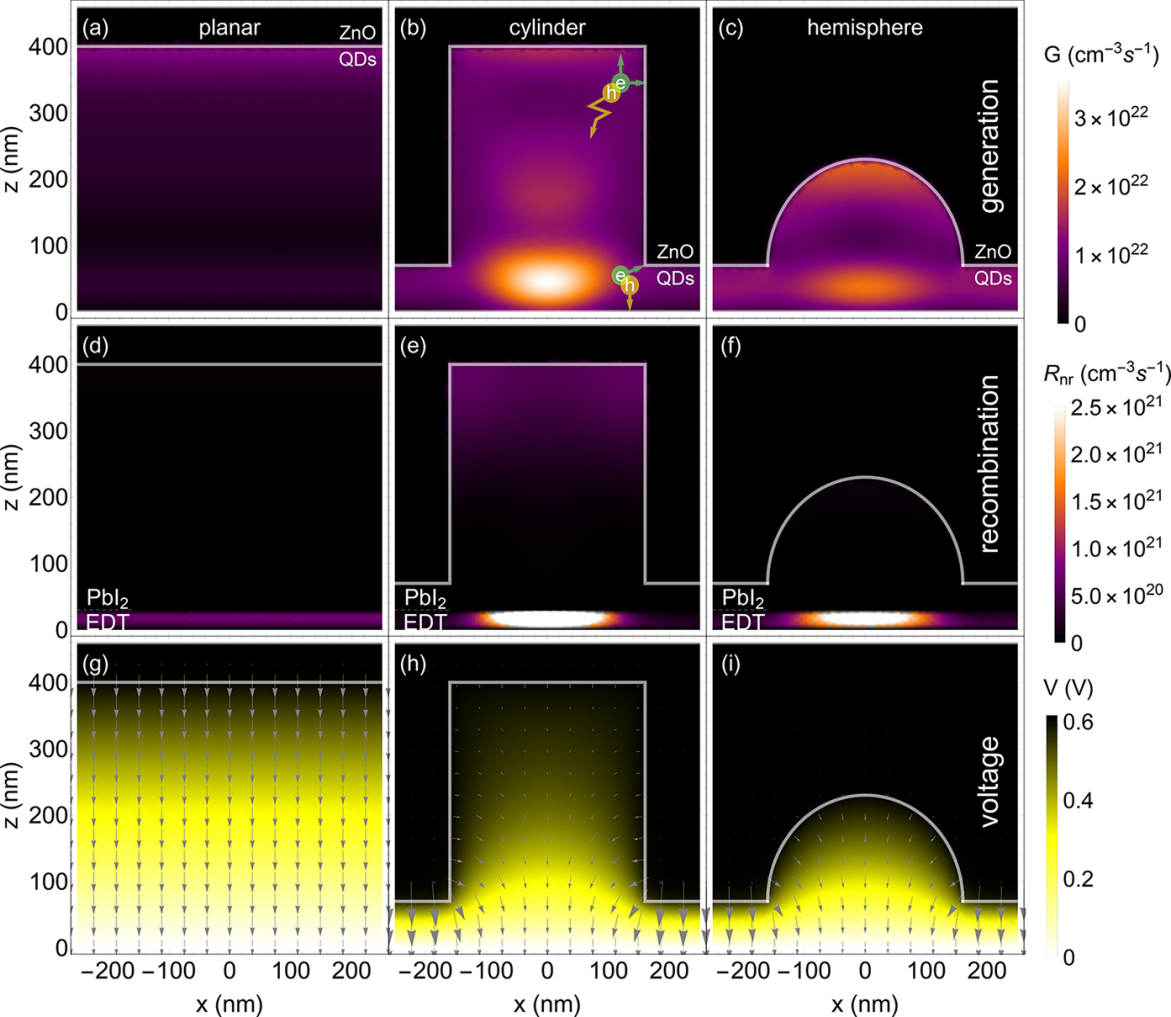
(a–c) Charge-carrier generation rates (*G*) across the cross-section of a (a) planar QD solar cell and a (b) cell with cylindrical and (c) hemispherical nanopattern. The color scale shows regions with high or low generation rates. (d–f) Nonradiative recombination rates (*R*_*nr*_) of charge carriers for the (d) planar, (e) cylindrical, and (f) hemispherical case. The interface between QD layers with different ligands is indicated by PbI_2_ and EDT. (g–i) Electric potential across p–i–n junction for (g) planar, (h) cylindrical, and (i) hemispherical patterned cases. The arrows show the electric field orientation and strength. Gray lines are used to show the interface between the ZnO and PbS QDs. A schematic of characteristic electron–hole pairs is shown in b.

We now investigate aspects of the differences in *J*_*SC*_, *V*_*OC*_, and *FF* for the three cases by investigating profiles of charge carrier generation (Figure 4a–c), recombination (Figure 4d–f), and potential gradients (Figure 4g–i). The strongly enhanced *J*_*SC*_ for the patterned cell compared to the thin cell results from the fact that it absorbs light much better for the same volume due to resonant light absorption in the nanostructure. Given the QD volume of the patterned device is 45% of that of the thick reference cell (400 nm), the *J*_*SC*_ results indicate that the largely reduced volume comes with a much smaller relative difference in *J*_*SC*_ (10%). We also note that compared to the *J*_*SC*_ that is directly calculated from the absorption spectrum, the actual *J*_*SC*_ value in the patterned case is slightly lowered, by 0.8 mA/cm^2^. This is due to some recombination occurring already at *V* = 0 (see Figure 4e).

The *V*_*OC*_ is similar for patterned and thin cells (704 mV and 697 mV, respectively) while in the thicker planar cell with the larger volume we find that the *V*_*OC*_ is lowered to 676 mV. The reason for this can be found in the static electric (E) fields across the absorber (Figure 4g–f). While the thick planar cell exhibits a maximum E-field strength (z-component) of 1.5 × 10^6^ V/m, the thin planar cell’s E-field goes up to 3.6 × 10^6^ V/m. This means that the thin cell can effectively resist external bias up to higher values and extract charges at larger applied voltages. In the case of the patterned cell, the E-field strength is well below 1.5 × 10^6^ V/m around the top of the pillar, but it increases significantly toward the bottom, also reaching around 3.6 × 10^6^ V/m in the very center, and even larger values toward the edges. We note that while we do not show simulated dark-IV curves here, their comparison leads to the same conclusions as this *V*_*OC*_ comparison.

So far, we have found that the nanopattern has two distinct advantages. First, it strongly enhances light absorption, which enables the use of less absorber material and increases the current, and second, the electric field strength is comparable to that of the thin cell, which leads to an optimized *V*_OC_. At the same time, the FF of the patterned cell is strongly reduced in these simulations. To find the origin of this, we first have to consider the generation profiles shown in Figure 4a–c, then consider Figure 4d–f to evaluate the associated recombination profiles, and then will find the connection between generation and recombination by considering the potential gradients in Figure 4g–i.

The generation profiles in Figure 4a–c are derived from electric field distributions such as those shown for three selected wavelengths in Figure 2e–g, integrating profiles over the full wavelength range until the bandgap. For the planar case (Figure 4a), the generation rate gradually decreases with depth, according to the Beer–Lambert law. In the patterned cell, most of the generation occurs at the bottom of the center of the nanopillar. The hotspots in the generation profile toward the bottom of the cell correspond mostly to absorption of light close to the bandgap that creates a well-defined resonant mode. The reason why this hotspot is found so close to the Au backcontact is that close to the backcontact incident and reflected light interfere more strongly with each other. While this creates a hotspot-like shape in the patterned case, this behavior can also be found at the back of planar solar cells, where usually the carrier generation increases again, deviating from a classical Beer–Lambert law absorption profile. This deviation can be understood as a sum of the incident light’s Beer–Lambert profile and the profile of the light reflected from the Au interface, which has highest values at the back of the cell and a decreasing exponential tail toward the front. The region of high generation at the top of the pillar corresponds to Beer–Lambert-like absorption of more energetic light.

In QD solar cells, charges that are generated in the vicinity of interfaces, within the depletion zone, have significantly higher chance of extraction and thus do not cause losses to the *J*_*SC*_. In the nanopatterned cell, generated electrons are on average much closer to the ZnO carrier collection interface that surrounds the nanopillars than in the case of the thick cell where carriers have to travel to the top or bottom interface. Effectively, the nanopatterned structure forms a bulk heterojunction geometry similar to that in other bulk heterojunction quantum dot (/organic, /dye-sensitized) solar cells, where tailored nanogeometries reduce the distance that charge carriers have to travel to the extraction interfaces and result in carrier generation in regions with stronger extraction fields. Furthermore, most holes are generated much closer to their extracting interface (bottom part of Figure 4b) (QD EDT – QD PbI_2_) than in the planar case, where most generation happens in the front. The schematic drawings of electrons (green) and holes (yellow) in Figure 4b indicate the required directions in which charges need to migrate.

Figure 4d (planar) and e (patterned) show where most of the nonradiative recombination occurs in the two cell types. For all cells, recombination is highest in the back due to poor carrier mobilities in the PbS-QD EDT layer. Besides that, for the planar case, recombination occurs homogeneously through the QD layer. In the patterned case, enhanced recombination occurs in the top region of the pillar, increasing radially away from the bottom center of the pillar.

To understand why this region is exposed to so much nonradiative recombination, the internal potential distribution for the two cell types is shown in Figure 4g and h. For the planar case (Figure 4e), we find a gradient across the depletion region, which extends across the whole absorber. In contrast, the gradient in the patterned case is radially aligned from the ZnO interface to the base of the pillar. The top region of the pillar shows only a very small potential gradient, explaining the strong recombination in that area. This explains the origin of the low *FF* in the patterned cells. For increasing cell voltages, the internal field decreases, and the weak-E-field region grows in volume, from the top of the pillar downward, and hence, recombination increases strongly with applied voltage, yielding a lower collected current density (Figure 3). This is equivalent to stating that the depletion region narrows with applied voltage and an increasing portion of charges is generated outside the depletion region and thus not contributing to the collected current.

This behavior can already affect the extracted current at *V* = 0. We use the simulated absorption spectrum from optical simulations and weigh it with the AM1.5G spectrum to obtain an upper bound for *J*_*SC*_ based on optical simulations. The ratio between this current and the current obtained from the electronic simulations yields the extraction efficiency of generated charges. Here, we define the internal quantum efficiency (*IQE*) for the absorber layer only, i.e., considering only photons that are absorbed by the absorber (PbS-PbI_2_, PbS-EDT) and neglecting parasitic absorption by the ITO and Au layers. The extraction efficiencies for 400 nm planar, 180 nm planar, and the patterned case are 99.5%, 97.5%, and 95.0%, respectively. This confirms the reduced extraction capabilities of the patterned structure at *V* = 0. Furthermore, the discrepancy between 400 and 180 nm planar layers implies that charges generated in the PbS-EDT layer at the back (relatively more generation happening there in the 180 nm case) are more likely to be lost to recombination than to be extracted. This suggests that charge carrier generation should ideally fully happen in the PbS-PbI_2_ layer, where charges can be separated more easily with the help of stronger electric fields (Figure 3a).

**Figure 5:**
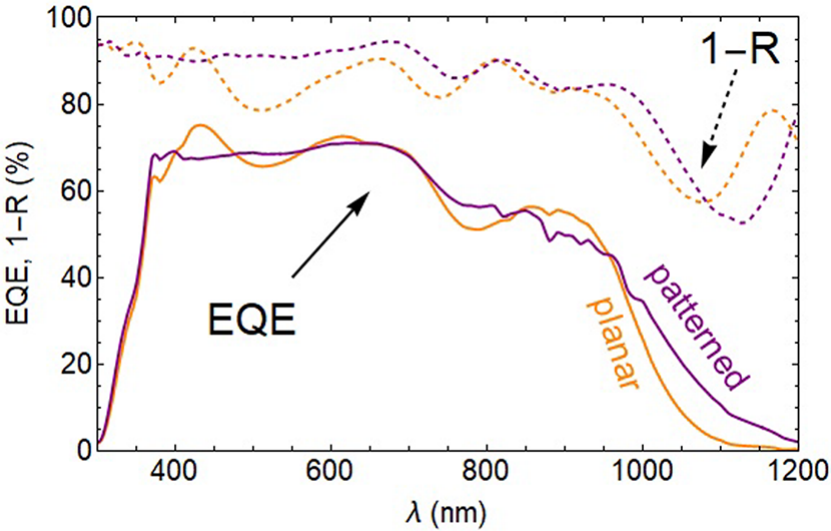
External quantum efficiency (*EQE*) and reflection losses (1–*R*) of a fabricated planar (orange) and patternd (purple) PbS QD solar cell, as a function of wavelength. Solid lines describe *EQE* curves and dashed lines correspond to the upper limits for the EQE, given by the cell reflection and represented by 1–*R*.

Experimentally measured *I*–*V* curves (Supporting Information) serve as further evidence regarding the subtle interplay between local fields, absorption/generation distance to the charge separating/collecting interfaces, and charge recombination. First of all, the increased *J*_*SC*_ confirms the enhanced light trapping observed in EQE measurements (Figure 5) and in simulations (Figure 2). Second, the reduced *FF* confirms the reduced carrier collection due to less optimal carrier extraction in the patterned case. Third, it is notable that the additional surface area does not create additional recombination as shown by the unchanged *V*_*OC*_ of the patterned cell.

In summary, the strong E-field at the pillar bottom allows for a larger *V*_*OC*_, while the weak E-field at the top induces the low *FF* due to extraction losses caused by holes that do not get extracted at the bottom of the cell. This realization immediately inspires further advanced cell designs that decouple the beneficial effects of local charge generation and collection near the collection interfaces and the detrimental effect of enhanced carrier recombination in field-free regions of the cell. For example, one could consider using a hemispherical shape (Figure 4c,f,i) instead of the pillar feature to create optical resonances. This would avoid weak-E-field regions at the top (Figure 4i), while the light absorption per unit volume is still enhanced (Figure 4c). In fact, by comparing expected *J*_*SC*_ values from optical and electronic simulations, we can get an extraction efficiency of 95.9% for this initial structure guess, which is already 0.9% higher than for the cylindrical structure. With respect to absorption, such a structure might come very close to a cylinder with an aspect ratio of 1 due to the similarity of the shapes. This would essentially correspond to cutting off part of the top of the cylinder that our optical optimization yielded in Figure 2c. As our optical optimization found different parameters for the cylinder dimensions than for those of the hemisphere, the hemisphere would come with some absorption loss relative to the optimized cylinder.

One option to circumvent this trade-off would be the introduction of an electronically inert material in place of the top half of the cylinder. With a real part of the refractive index similar to that of the QDs, and with a negligible imaginary part, this material would deliver the same optical resonances, but with essentially all generation happening in the bottom half of the cylinder. This would solve the issue of the low *FF* while keeping *V*_*OC*_ and *J*_*SC*_ at similarly high values.

Another potential option to tackle the weak extraction from the top of the cylinder would be the utilization of a graded bandgap, which is a well-investigated concept for PbS QD solar cells.^45^ By having a few layers of QDs with consecutively smaller bandgaps toward the top of the cylinder (the bandgap would approach the desired overall bandgap toward the bottom), a gradient would emerge in the conduction and valence bands due to the bandgap shrinking toward the top. This would aid with directing the diffusing carriers in the right direction. Naturally, such a structure would have a lower *V*_*OC*_ due to the addition of QDs with slightly smaller bandgaps. But this loss might be overcome by a significantly increased *FF*.

In general, the observations made so far can potentially help bring the efficiency of PbS quantum dot solar cells beyond current record power conversion efficiencies. So far, one of the main goal for PbS QD solids within the scientific community has been the improvement of the charge carrier diffusion length.^47^ While this is an important objective, our simulations outline an alternative road toward highly efficient solar cells. Better control over the charge carrier generation profile could potentially allow for a weakening of the constraints that are imposed by short diffusion lengths. Generating charges closely to extraction interfaces requires shorter average charge migration and hence less susceptibility to carrier recombination. We have also shown that there is no benefit if the distribution of the electric fields is affected negatively by the structured absorber. More strongly doped p- and n-type layers^46^ could potentially help with mitigating the effect of weakened electric fields due to structuring, and lead to overall gains, if diffusion lengths in these materials are much lower than in the materials used in this study. If the diffusion length of the absorber is much longer, then the charge extraction is also improved for the patterned case. This favors shapes that strongly interact optically, as electronic constraints are alleviated. In this case, the structure is more competitive compared to thicker planar cases, as the strong optical performance is coupled with lower extraction losses. Finally, the results so far put emphasis on the role of shapes within the device architecture, and while fabrication of planar structures is in many cases easier, there is no obvious reason why the ideal solar cell geometry should consist of such a highly planar geometry.

### Electronic Characterization of Patterned Junction

Finally, we characterize the electronic performance of a patterned and planar QD solar cell by measuring the wavelength-resolved external quantum efficiency (*EQE*). Figure 5 shows a comparison between *EQE* and light transmitted into the cell (1–*R*) for thick planar and patterned cells. In the initial experiments, the QD pillars have a radius of 190 nm, a height of 100 nm, a pitch of 513 nm, and the residual layer is 400 nm thick. The *EQE* represent the number of electrons and holes that arrive at the metal contacts and are extracted, relative to the incident photon flux. The *EQE* of the planar device clearly shows the first exciton peak (900 nm), as well as two Fabry-Perót cavity features (450 and 650 nm). For the patterned device, we can identify the first exciton peak at the same wavelength, but no Fabry-Perót resonances are distinguished. A flat *EQE* spectrum is found for wavelengths shorter than 700 nm. Importantly, we find a considerably higher *EQE* toward the bandgap (950–1200 nm) for the patterned case. The reflection spectra confirm those features, except for the range around the first exciton peak.

Several conclusions can be drawn from the *EQE* results. First of all, the comparison of the two *EQE* spectra shows that the complex nanopatterning procedure to fabricate these QD bulk heterojunction cell geometry yields devices with better *EQE* than the planar cells, demonstrating the applicability of SCIL to fabricate good-quality devices.

Second, the IR response (1000–1200 nm) is significantly better in the patterned case. This is indicative of longer effective path length that light experiences within the absorber layer and in good agreement with the wavelength dependent enhancement presented in Figure 2b, which is highest close to the bandgap, similar to the *EQE* measurement.

Third, in both cases, the *EQE* and reflection loss align well with each other for wavelengths below 750 nm. The gap between *EQE* and (1–*R*) curves corresponds to charges that are lost either to parasitic absorption or to extraction losses. For wavelengths around the first exciton peak, the *EQE* and (1–*R*) data have a bigger gap, indicating that the charges generated by lower energy light are extracted less efficiently. We attribute this to generation of charges in regions that are too far away from extracting interfaces. Alternatively, the light may be lost to parasitic absorption in the ITO layer.

We note that the experimental geometry used here is quite different than the optimum obtained from simulations. Notably, the pillar height (100 nm vs 330 nm for the optimum) and residual thickness (400 nm vs 70 nm), and further enhancements can be expected in further improved experiments.

From the reflection spectra, we derive a maximum enhanced *J*_*SC*_ gain of 2.00 mA/cm^2^ for the nanopatterned cell. However, the integrated *EQE* spectra indicate a *J*_*SC*_ gain of 0.74 mA/cm^2^ for the patterned case. The difference, 1.26 mA/cm^2^, is lost by incomplete extraction or to parasitic absorption. This incomplete extraction is further confirmed by an IV-analysis (Supporting Information), which shows that experimental planar and patterned cells suffer from a lower FF than in the simulations, associated with more strongly decreasing charge extraction with increasing voltage. Overall, the *EQE* shows a higher *J*_*SC*_ for the patterned case, mainly owing to mitigation of Fabry-Perót cavity related dips in the short wavelength region and a better EQE response around the bandgap (1000–1200 nm) due to effective path length enhancement for near-infrared photons.

## Conclusions

Optically resonant heterojunctions can benefit the charge collection of solar cells with short carrier diffusion lengths in addition to the advantages of enhanced light incoupling and trapping. We demonstrate a nanopatterned p–n junction for a QD solar cell and present a method for fabrication of such structures using soft nanoimprint. We then use optical simulations to maximize the absorption per unit volume in a QD solar cell and significantly increase absorption in comparison to a reference structure. Additionally, the optical optimization yields a charge carrier generation profile that is vastly different from the conventional Beer–Lambert law-like profile of planar cells.

The impact of such a pattern on the IV characteristics of a device is investigated in electronic simulations and indicates that such structuring allows for both improved *J*_*SC*_ and *V*_*OC*_ values. Our initial design shows a reduced *FF*, which is attributed to inhomogeneous electric field distribution across the absorber that leads to locally weaker charge extraction for increased voltage. For the same absorber volume, an efficiency gain of 0.4% is found in the patterned case.

Based on these insights, we suggest two pathways for further improving the efficiency by dealing with the low *FF*. One option entails introduction of an electronically inert material with similar optical properties as the QDs, that serves as an effective optical antenna, while the generation of charges occurs closer to the collecting contact layers. The other option would utilize a graded bandgap structure to direct diffusing charges toward the space charge region from where they can be extracted more easily.

Finally, we show experimental results for a nanopatterned heterojunction design and find that the *EQE* spectrum of the patterned cell differs considerably from the planar case, corresponding to an enhanced *J*_*SC*_ of 0.74 mA/cm^2^. Overall, our work shows a solar cell architecture that optimizes light management and charge carrier extraction. These observations are independent of the absorber material and apply also to other materials with short diffusion lengths.

## Methods

### ZnO Nanoparticle Synthesis

The synthesis of zinc oxide (ZnO) nanoparticles was performed according to previously published methods.^31^ For this, 2.95 g of zinc acetate dihydrate (Zn(Ac)_2_·2H_2_O) was dissolved by 125 mL of methanol at 60 °C and the solution was kept stirring at 60 °C. Then 1.48 g of potassium hydroxide (KOH) was dissolved by 65 mL of methanol. The KOH-methanol solution was added to the Zn(Ac)_2_ solution drop-by-drop. Next, the solution was kept at 60 °C and stirred for 2.5 h. The product was precipitated by centrifugation and washed by using methanol twice. The washed ZnO nanoparticles were dissolved by 10 mL of methanol/chloroform (1:1 v/v).

### ZnO Patterning Using SCIL

For the patterned ZnO NP layer, commercial glass substrates with 200 nm ITO were cleaned by sonicating for 10 min each in detergent, acetone, and IPA. Then the ZnO NPs solution was dropped onto the ITO substrate and the solution was spread and thinned briefly using a spin-coater. After 3.5 s at 2000 rpm, the sample with the thin liquid ZnO layer was taken out of the spin-coater and the PDMS stamp was applied onto it to transfer the nanopattern into the ZnO NP layer. After 6 min of room temperature curing, the stamp was peeled off, which concluded the patterning process.

### PbS Synthesis

PbS colloidal quantum dots (CQD) were synthesized according to previously published methods. First, 460 mg of lead(II) oxide (PbO, 99.999%, Sigma-Aldrich) was mixed with 10 mL of octadecene (technical grade, Sigma-Aldrich) and 2 mL of oleic acid (technical grade, Sigma-Aldrich). The solution was heated to 120 °C and degassed for 1 h. Then the temperature was reduced to 90 °C and 5 mL of octadecene solution with 140 μL of hexamethyldisilathiane (TMS_2_S) (synthesis grade, Sigma-Aldrich) was injected swiftly. After the injection, heating was turned off and the solution was allowed to cool to room temperature gradually. The as-synthesized CQD-solution was purified by adding acetone (VWR) before centrifugation at 6000 rpm for 5 min, followed by redispersing with hexane (95%, Sigma-Aldrich). This washing process was repeated twice, and the precipitate was dispersed by octane (50 mg/mL).

### PbS Ink Preparation

PbS ink was prepared by using previously published solution ligand-exchange process.^48^ For this, 553.2 mg of lead(II) iodide (PbI_2_), 174.1 mg of lead(II) bromide (PbBr_2_), and 55.5 mg of ammonium acetate (NH_4_Ac) were dissolved by 12 mL of *N*,*N*-dimethylformamide (DMF). Next, 7.5 mL of 10 mg/mL PbS-octane solution was added into the solution. The mixture was shaken by using a vortex mixer for 5 min. The upper layer of the solution was removed. The solution was washed in octane 2–3 times. Then toluene was added into the solution followed by centrifuging at 6000 rpm for 5 min to precipitate the quantum dots. The precipitate was dried by vacuum and dispersed by butylamine. The final concentration was ∼300 mg/mL.

### PbS Spin-Coating

For spin-coating, 40 μL of PbS ink was applied on substrates followed by spin-coating at 1000 rpm for 3 s, and then 2000 rpm for 30 s. The film was annealed at 80 °C for 2 min.

### PbS QD EDT layer and Au layer

Afterward, two thin PbS QD OA layers of 15 nm each were deposited, and each was subjected to solid-state ligand exchange to obtain PbS QD EDT (ethanedithiol) layers. The back-contact was fabricated by evaporation of Au at an initial rate of 0.01 nm/s for the first 10 nm and then 0.1 nm/s to get to 100 nm final thickness.

### Ellipsometry

The required samples were prepared by spin-coating thin layers (30–150 nm) of PbS QDs onto Si substrates. Subsequently, spectroscopic ellipsometry (SE) data sets were taken at angles of incidence of 60–80° using a Woolam Ellipsometer. The spectra were analyzed using CompleteEASE. A Cauchy model was used to obtain the thickness and refractive index of the QD layer in the infrared (IR) range where absorption was negligible. After fixing these values, the Cauchy model was replaced by a b-Spline model and initially fitted for the nonabsorbing wavelength range in the IR only. Then the fitted range was incrementally increased toward the UV to get a first approximation of the optical constants, according to the used b-Spline model. That model was then replaced by a Gaussian oscillator model. As the inhomogeneous broadening is much stronger than the homogeneous broadening^49^ for the first exciton peak, it is modeled by a Gaussian. The onset of the continuous, bulk PbS part of the absorption spectrum is also modeled by the tail of a Gaussian. For PbS QD films with OA ligands, a third oscillator is included as that greatly improves the fit accuracy.

### Optical Simulations: Lumerical FDTD

Except for the QD layers and the ITO layer, optical constants from the Lumerical FDTD library were used for ZnO and Au. After convergence testing, the simulation mesh accuracy was set to “5” and “conformal variant 1”. Symmetric/antisymmetric boundary conditions were employed along the cell’s plane and perfectly matched layers (PML) were used at the top and bottom of the simulation geometry.

### Electronic Simulations: Lumerical CHARGE

The simulation geometry was described by a triangular mesh, which was sufficiently fine to accurately describe all features of the simulated device. Planar structures were simulated in 2D, patterned structures in 3D. Parameters for the p-, i-, and n-layers are listed in Table 1 and in the Supporting Information. Both contact layers were defined as Ohmic contacts with parameters specified in Table 2. For performance under AM1.5 solar irradiation, a charge carrier generation profile was imported from Lumerical FDTD, using the dedicated “Solar Generation” function. To obtain an *I*–*V* curve, the simulation geometry is evaluated separately for a range of voltage values across the relevant voltage range. For simplicity, recombination processes that have not received much attention in recent years (interface recombination of PbS QDs and ZnO) have not been included in the simulations. Depending on the interface structure/roughness, this might lead to minor gains or losses in the overall performance,^50^ but the literature does not provide reliable values that could be used in the simulations.

**Table 1:** List of Parameters^18,25,41−45^ Used for Semiconductors in 3D Drift-Diffusion Simulations in Lumerical CHARGE

quantity	label in software	unit	ZnO	PbS-PbI_2_	PbS-EDT
DC permittivity	DC permittivity		66	20	20
Work function	Work function	eV	5.95	4.7	4.55
High symmetry point in bandstructure with lowest conduction band valley	Ec valley		Γ	Γ	Γ
Effective electron mass	Effective mass mn	1/m_e_	0.24	0.54	0.54
Effective hole mass	Effective mass mp	1/m_e_	0.59	0.54	0.54
Bandgap	Eg	eV	3.3	1.1	1.1
Electron mobility	Mun	cm^2^/(Vs)	100	0.02	0.0002
Hole mobility	Mup	cm^2^/(Vs)	25	0.02	0.0002
Nonradiative electron lifetime	Taun	s	1.0 × 10^–9^	2.4 × 10^–7^	2.4 × 10^–7^
Nonradiative hole lifetime	Taup	s	1.0 × 10^–9^	2.4 × 10^–7^	2.4 × 10^–7^
Trap state energy level offset	Ei offset	eV	1.1	0.4	0.4
Optical capture rate coefficient	Copt	cm^3^/s		5 × 10^–13^	5 × 10^–13^
Donor doping density	ND:n	cm^–3^	10^18^	10^15^	10^14^
Acceptor doping density	NA:p	cm^–3^	0	10^15^	10^17^

**Table 2:** List of Parameters^18,25,41−45^ Used for Semiconductor Metal Interfaces in 3D Drift-Diffusion Simulations in Lumerical CHARGE

quantity	label in software	unit	Au–PbSEDT interface	ITO–ZnO interface
Metal workfunction	Workfunction	eV	5.1	4.7
Interface recombination velocity	SRV	cm/s	1.0 × 10^9^	1.0 × 10^9^

### EQE and Reflection Measurements

We used a PV Measurements QEX7 system for the EQE measurements. The spot size of the incident light beam was 1 mm × 4 mm and smaller than the contact area, which was necessary to not loose charges generated in nonactive areas. In parallel, we measured reflection spectra for the same samples, with a PerkinElmer1050 tool. The samples were fixated at the backside of an integrating sphere, and the reflection spectrum was collected for an angle of incidence of 8° to include specular reflection. For this step, Au was evaporated over the full backside area of the cell.

## Supporting Information Available

Further details about experimental analysis of fabricated cells: current–voltage curves under 1-sun illumination, statistical comparison of electronic parameters of fabricated patterned and planar solar cells (PDF)
