# Supplementary material for: Optically Resonant Bulk Heterojunction PbS Quantum Dot Solar Cell
Source: ACS Nano. 2022 Aug 29;16(9):13750–60. doi: 10.1021/acsnano.1c11330 (PMC9527793; doi:10.1021/acsnano.1c11330)
Supplement: Supplementary file 1 — nn1c11330_si_001.pdf [file nn1c11330_si_001.pdf]

Supporting info

Optically resonant bulk heterojunction PbS quantum dot solar cell

Stefan W. Tabernig[1,2,*], Lin Yuan[2,4], Andrea Cordaro[1,3], Zhi Li Teh[2], Yijun Gao[2], Robert J. Patterson[2], Andreas Pusch[2], Shujuan Huang[4], Albert Polman[1,*]

[1] Center for Nanophotonics, NWO-Institute AMOLF, Science Park 104, 1098 XG Amsterdam, The Netherlands
[2] School of Photovoltaic and Renewable Energy Engineering, University of New South Wales, 229 Anzac Parade, 2052 Sydney, Australia
[3] Van der Waals-Zeeman Institute, Institute of Physics, University of Amsterdam, Science Park 904, 1098 XH Amsterdam, The Netherlands
[4] School of Engineering, Macquarie University, Sydney 2109, Australia

Light current-voltage curves of fabricated samples

Figure S1 shows a current density - voltage (*J-V*) curve comparison between fabricated planar and patterned PbS QD solar cells under 1-sun AM1.5G illumination. The pattern dimensions are the same as stated in the main manuscript (section: Electronic Characterization of the Patterned Junction), and the fabrication details can be found in the Methods section. Different solar cells were fabricated for the light-*J-V* measurements than for the *EQE* characterization, as *J-V* measurements require smaller Au backcontacts (100-nm-thick Au disks with 1 mm radius), to minimize the impact of fabrication defects on the electronic properties. Both cells originate from the same batch, and every cell contains 9 subcells with distinct backcontacts.

The trends for short-circuit density (J_{SC}), open-circuit voltage (V_{OC}), fill factor (*FF*), and efficiency (η) observed in Figure S1 correspond well with the trends predicted by simulations (Figure 3b). The J_{SC} is increased significantly. The average V_{OC} shows a slight gain within the error bar range. The *FF* is reduced in the patterned case. The efficiency shows a slight gain within the error bar range.

We note that the observed trends are sometimes confined to the error bar range. Furthermore, the planar reference cell exhibits a fairly low *FF*. To investigate the quality of the baseline and the degree to which the data shown in Figure S1 is representative, we fabricated a separate batch of planar samples (Figure S2). This batch was fabricated after the batch that yielded the comparison between planar and patterned samples, and shows improved electronic parameters. However, we expect that the conclusions on the sample-to-sample variation are equally valid.

The investigation of the baseline shows that the difference in the electronic parameters from cell-to-cell can be larger than the error bars that are derived from individual subcells. One reason for this is that the Au backcontact area is associated with an error of up to 20% for the Au disk radius, due to variations in evaporation mask procedure . This affects the J_{SC} as it is calculated by normalising the measured current to the backcontact area. Furthermore, it was difficult to control the quality and deposition conditions (air flow) of the PbS QD ink precisely, which affected the electronic quality of the cells.

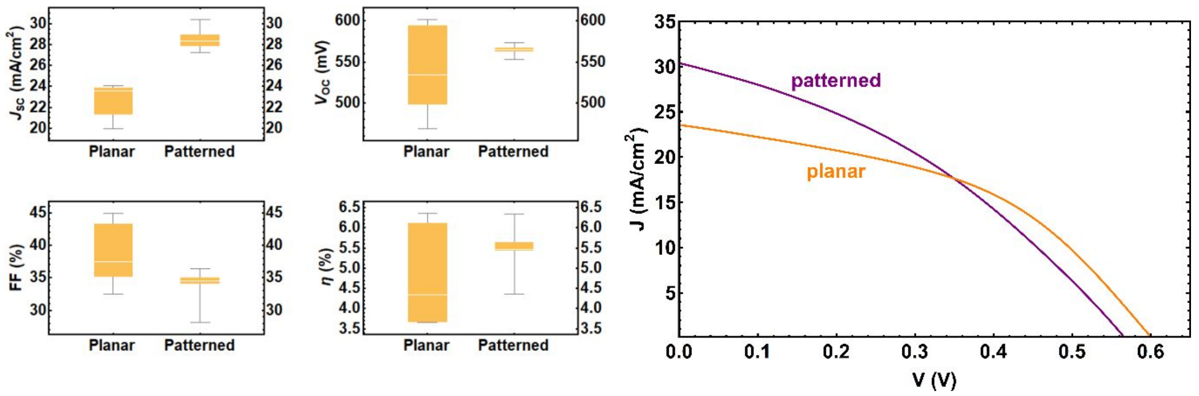

Figure S1: Left: Box-plot diagrams of short-circuit current density (J_{SC}), open-circuit voltage (V_{OC}), fill factor (*FF*) and efficiency (η) of a planar and a patterned solar cell. Each cell contains 9 subcells. Right: *J*-*V*-curves of the best planar (orange) and patterned (purple) subcell.

The difference in J_{SC} in the patterned-planar comparison (Figure S1) is larger than the baseline sample to sample variation observed in Figure S2. The differences in V_{OC} and *FF* in the patterned-planar comparison are within the baseline sample-to-sample variation observed in Figure S2. This suggests that the impact of the patterned structure on the electronic properties (V_{OC}, *FF*) is not very detrimental. Finally, the efficiency statistics do not allow for identifying a superior geometry, but do confirm that the realisation of a nanopatterned p-n junction yields properly functioning electronic devices.

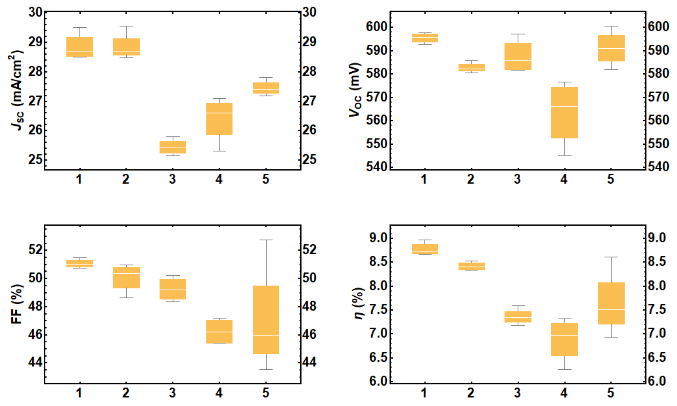

Figure S2: Box-plot diagrams of short-circuit current density (J_{SC}), open-circuit voltage (V_{OC}), fill factor (*FF*) and efficiency (η) of five planar solar cells from a single fabrication batch. The numbers on the x-axis denote different cells, with 9 subcells each.
